# Genetic Diversity of *Plasmodium vivax* Field Isolates from the Thai–Myanmar Border during the Period of 2006–2016

**DOI:** 10.3390/tropicalmed8040210

**Published:** 2023-03-31

**Authors:** Abdifatah Abdullahi Jalei, Wanna Chaijaroenkul, Kesara Na-Bangchang

**Affiliations:** 1 Chulabhorn International College of Medicine, Rangsit Campus, Thammasat University, Pathum Thani 12121, Thailand; 2 Drug Discovery and Development Center, Rangsit Campus, Thammasat University, Pathum Thani 12121, Thailand; 3 Center of Excellence in Pharmacology and Molecular Biology of Malaria and Cholangiocarcinoma, Chulabhorn International College of Medicine, Rangsit Campus, Thammasat University, Pathum Thani 12121, Thailand

**Keywords:** *Plasmodium vivax*, genetic diversity, *PvCSP*, *PvMSP-3*, Thai–Myanmar border

## Abstract

High levels of genetic variants of *Plasmodium vivax* have previously been reported in Thailand. Circumsporozoite surface protein (CSP), merozoite surface protein (MSP), and microsatellite markers were used to determine the genetic polymorphisms of *P. vivax*. This study aimed to investigate the molecular epidemiology of *P. vivax* populations at the Thai–Myanmar border by genotyping the *PvCSP*, *PvMSP-3α*, and *PvMSP-3β* genes. Four hundred and forty *P. vivax* clinical isolates were collected from the Mae Sot and Sai Yok districts from 2006–2007 and 2014–2016. Polymerase chain reaction with restriction fragment length polymorphism (RFLP) was used to investigate the genetic polymorphisms of the target genes. Based on PCR band size variations, 14 different *PvCSP* alleles were identified: eight for VK210 and six for VK247. The VK210 genotype was the dominant variant during both sample collection periods. Based on PCR genotyping, three distinct types (A, B, and C) for both *PvMSP-3α* and *PvMSP-3β* were observed. Following RFLP, 28 and 14 allelic variants of *PvMSP-3α* and 36 and 20 allelic variants of *PvMSP-3β* with varying frequencies were identified during the first and second periods, respectively. High genetic variants of *PvMSP-3* and *PvCSP* were found in the study area. *PvMSP-3β* exhibited a higher level of genetic diversity and multiple-genotype infection versus *PvMSP-3α*.

## 1. Introduction

Malaria is one of the most important infectious diseases worldwide. *Plasmodium vivax*, one of the five *Plasmodium* species, is the most common cause of malarial morbidity in Asia, Central America, and South America [1,2]. About 90% of malaria cases in Thailand are caused by *P. vivax* [3]. In recent years, the proportion of *P. vivax* malaria cases in the districts or towns of Thailand that border Myanmar, Malaysia, Cambodia, and Laos has increased [4]. Malaria control depends mostly on antimalarial drugs and insecticides [5], and vaccines are seen as a possible additional way to fight malaria [6]. Thus, the widespread presence of multidrug-resistant parasites has a significant effect on malaria control [7]. Studies on the genetic diversity of the parasite population are important to understand how parasite virulence has evolved over time and the role of parasite diversity in malaria transmission. This information provides insight into how the parasite population may respond to interventions [8,9]. *P. vivax* exhibited a significantly higher level of genetic polymorphism than *P. falciparum* [10]. The complex interactions between parasites, parasites and hosts, parasites and vectors, and geographic regions are the major factors that affect the genetics of *P. vivax* [11]. Microsatellites, as well as genes encoding the circumsporozoite protein (CSP) and merozoite surface protein-3 alpha and beta (MSP-3α and MSP-3β), are among the polymorphic genetic markers used to study the genetic diversity of *P. vivax* in malaria-endemic areas [12,13]. *PvCSP* is a highly immunogenic sporozoite surface polypeptide that is considered to be a potential malaria vaccine candidate [14]. Based on variations in the sequences and numbers of peptide repeat motifs in the central repetitive region of *PvCSP*, three distinct genotypes (VK210, VK247, and *P. vivax*-like) were found [15]. These genotypes are found worldwide, with VK210 being the most prevalent in many endemic areas [16]. The *PvMSP-3* multigene family consists of several related proteins, including *PvMSP-3α*, *PvMSP-3β*, and *PvMSP-3γ*. Of these, *PvMSP-3α* and *PvMSP-3β* are highly polymorphic and are regarded as potential vaccine candidates [17]. Understanding their genetic diversity is therefore important, because variations in candidate vaccine antigens can hamper their efficacy [4]. High levels of genetic diversity at these loci have been reported from diverse geographic areas of Thailand; 33 and 20 alleles of *PvMSP-3α* and 28 and 13 alleles of *PvMSP-3β* have been reported from the Thai–Myanmar border and the western region of Thailand, respectively [4,18]. Therefore, understanding the genetic diversity of Thai *P. vivax,* which has been circulating in the country over time, can provide evidence for the association of certain genotypes with disease epidemiology and pathogenesis. The current study aimed to investigate the genetic diversity of *PvCSP*, *PvMSP-3α*, and *PvMSP-3β* in clinical isolates from the Thai–Myanmar border.

## 2. Materials and Methods

### 2.1. Ethical Approval

The study protocol was approved by the Ethics Committee of Thammasat University (No. 082/2560). Informed consent was obtained from each participant before sample collection.

### 2.2. Sample Collection and Plasmodium Diagnosis

A total of 440 *P. vivax*-positive blood samples were collected during two different periods (2006–2007 and 2015–2016) from patients visiting hospitals or malaria clinics in the provinces of Tak and Kanchanaburi. In the first period, 330 samples were collected from patients in Mae Sot (Tak province) and Sai Yok (Kanchanaburi province), whereas the remaining 110 samples were collected from patients in Mae Sot district during the second period. The *Plasmodium* species were identified using Giemsa-stained smears. Blood samples were plotted on filter papers and used for molecular analysis.

### 2.3. Genomic DNA Extraction

Genomic DNA was extracted from dried blood spots using a QIAamp DNA Mini Kit (Qiagen, Valencia, CA, USA) in accordance with the manufacturer’s extraction protocol. The extracted DNA templates were used for parasite genotyping.

### 2.4. P. vivax Parasite Genotyping

Polymerase chain reaction with restriction fragment length polymorphism (PCR-RFLP) techniques were used to examine allelic variation in the *PvCSP*, *PvMSP-3α*, and *PvMSP-3β* genes using primers described elsewhere [19,20,21]. To distinguish the genotypes of the *PvCSP* gene, nested PCR products for each sample were separately digested with *Bst NI* and *Alu I* enzymes (New England Biolabs Inc., Hitchin, UK), following the manufacturer’s instructions. For *PvMSP-3α* and *PvMSP-3β* allelic variant assessments, the second PCR products were digested with *Hha I* and *Pst I* enzymes, respectively (New England Biolabs Inc.). The digested fragments were electrophoresed on a 2.0% agarose gel stained with Neogreen (Neo Science, Seoul, Republic of Korea), and restriction banding patterns were used to identify the alleles of each gene.

### 2.5. Statistical Analysis

Allele frequencies of *PvCSP*, *PvMSP-3α*, and *PvMSP-3β* were calculated as the proportion of alleles observed for each gene. A Chi-square test or Fisher’s exact test was used to compare allele frequency associations between the two periods using SPSS software version 21 (IBM Corp., Armonk, NY, USA). The statistical significance level was set at *α* < 0.05.

## 3. Results

### 3.1. Genetic Diversity of the PvCSP

**Figure 1:**
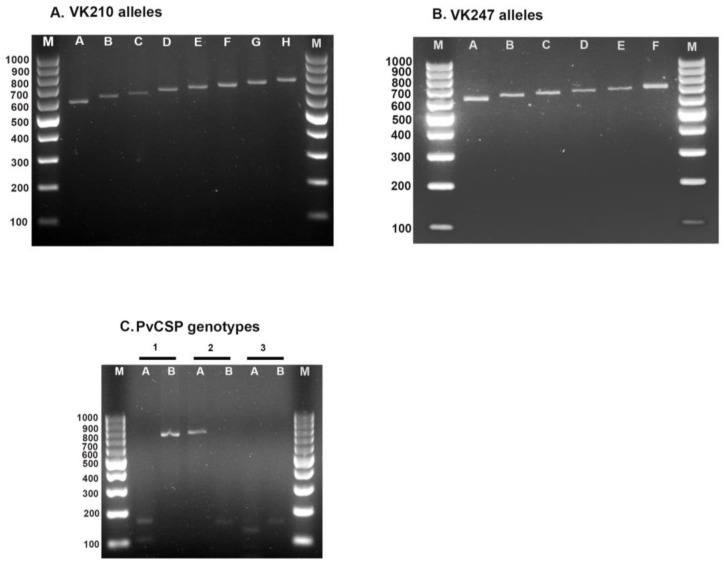
PCR product size variations and genotypes of *PvCSP.* Gel images depict: (**A**) PCR product size variations for VK210; (**B**) PCR product size variations for VK247; and (**C**) *PvCSP* products digested with *AluI* (A) and *BstNI* (B) enzymes. Sample No. 1: A cut the product into small fragments, but B did not cut the product (VK210 genotype). Sample No. 2: A did not cut the product into small fragments, but B cut the product (VK247 genotype). Sample No. 3: A and B cut the products into small fragments (mixed genotype). M: Molecular marker (100 bp).

The *PvCSP* gene was successfully genotyped in 417 of 440 *P. vivax* samples (94.8%). Of these, 315 of 330 (95.5%) and 102 of 110 (92.7%) isolates were from the first and second study periods, respectively. The RFLP of the *PvCSP* genotypes is shown in Figure 1C. When the two sample collection periods for *PvCSP* were compared, statistically significant differences were observed (*p* = 0.012). The results showed that the VK210 type was the most common (94.0% and 87.3% in the first and second sampling periods, respectively; Table 1). Interestingly, the frequency of the VK247 type increased during the second sampling period. Mixed infections with both genotypes, VK210 and VK247, were detected in only two isolates from Sai Yok. According to the size variation in the PCR bands, 14 different alleles (8 for VK210 and 6 for VK247) were found. The variations were arranged in an increasing pattern of 20 base pairs (bp). VK210 had a PCR band size range of 620–800 bp, and VK247 had a range of 620–740 bp (Figure 1A,B).

### 3.2. Genetic Diversity of the PvMSP-3α

**Figure 2:**
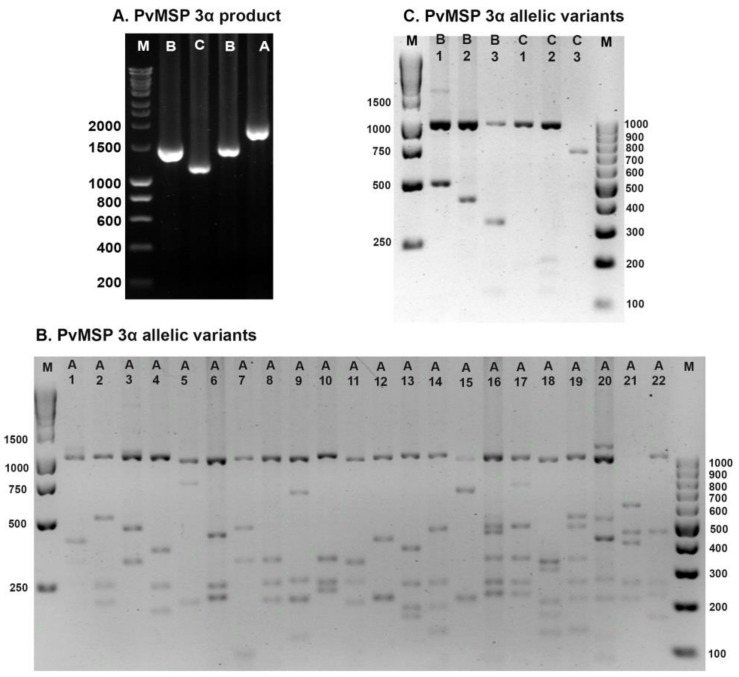
PCR product size variations and allelic variants of *PvMSP-3α.* Gel images depict: (**A**) PCR product size variations; (**B**) allelic variants for type A; and (**C**) allelic variants for types B and C. M: Molecular markers (100 bp and 1000 bp).

The *PvMSP-3α* was successfully genotyped in 373 (84.8%) of 440 samples. Of these, 277 of 330 (83.9%) isolates were from the first period, and 96 of 110 (87.3%) isolates were from the second period. Based on PCR band size variations for the *PvMSP-3α* gene, three distinct types, namely, types A (1.9 kb), B (1.5 kb), and C (1.1 kb) (Figure 2A) [18], were detected in the isolates collected during the first period, whereas types A and B were found in the isolates collected during the second period (Table 2). After *Hha I* digestion, most isolates showed a conserved 1000 bp band with varying patterns of small amplicons (Figure 2B,C). On the basis of these results, 21 allelic variants of type A and 3 allelic variants each of type B and type C were identified in the first sample collection period. Allele A8 was the most common allele in Sai Yok isolates (17.9%), whereas allele C1 was the most prevalent allele in Mae Sot isolates (14.4%). From all the samples analyzed for the first period, multiple-genotype infections were detected in 11 (4.0%) isolates when two different PCR bands were found in a single sample (5 isolates) or when the restriction analysis of a single PCR product exceeded the uncut PCR band size (6 isolates). The isolates from the second period contained 12 allelic variants of type A and 2 allelic variants of type B. Allele A11 was the most abundant allele, accounting for 32.3% of the samples. No mixed genotypes were detected during the second period. The overall differences in allele frequency between the two periods were statistically significant (*p* < 0.0001). There were significant differences between mixed genotype (*p* = 0.002) and B (*p* < 0.001). In addition, Mae Sot isolates from the two different time periods showed a statistically significant difference (*p* = 0.009).

### 3.3. Genetic Diversity of the PvMSP-3β

**Figure 3:**
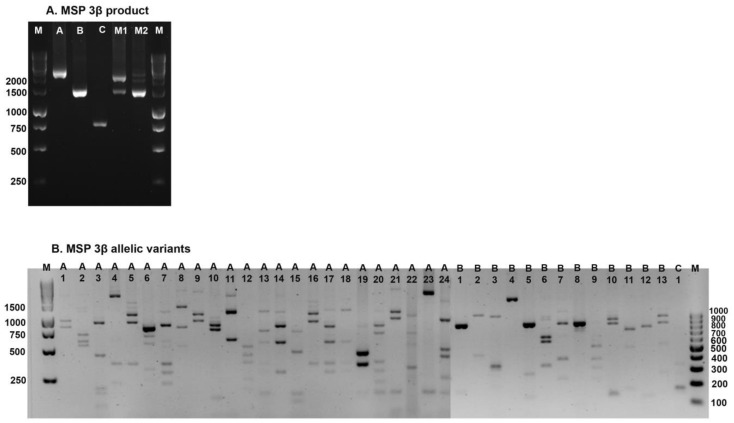
PCR product size variations and allelic variants of *PvMSP-3β.* Gel images depict: (**A**) PCR product size variations, and (**B**) allelic variants for types A, B, and C. M: Molecular markers (100 bp and 1000 bp). M1 and M2: Mixed genotypes.

The PvMSP-3β was successfully genotyped in 328 (74.5%) of 440 samples. Of these, 248 of 330 (75.2%) isolates were from the first period, and 80 of 110 (72.7%) isolates were from the second period. Based on PCR band size variations, three distinct types were identified in the first period isolates: type A (1.7–2.3 kb), type B (1.4–1.6 kb), and type C (0.780 kb) (Figure 3A) [19]. Only types A and B were observed in the second period. The majority of samples from the first period (63.4%) belonged to type A, whereas in the second period, types A and B were equally prevalent (Table 3). Different banding patterns were observed when PCR products were digested with *Pst I* enzyme (Figure 3B). The isolates from the second period contained 22 allelic variants of type A, 13 allelic variants of type B, and one allelic variant of type C. Allele A1 (~19.0%) was the predominant allele in Mae Sot and Sai Yok isolates. Different allelic patterns were observed in the two endemic areas during the first period; Sai Yok isolates showed higher genetic diversity than Mae Sot isolates (29 *vs.* 22 alleles). The isolates from the second period were classified into types A and B with 9 and 10 allelic variants, respectively. Allele B4 was the most prevalent allele, accounting for 27.5% of the isolates. Multiple-genotype infections of the first and second periods were observed in 43 (17.3%) and 11 (13.8%) isolates, respectively. This occurred when more than one PCR product of a different size was found in a single sample (15 and 1 isolates, respectively), or when the summed size of the DNA fragments resulting from the restriction digestion exceeded the size of the PCR product (43 and 11 isolates, respectively). There was no statistically significant difference in the frequency of the *PvMSP-3β* alleles between the two periods (*p* = 0.88).

## 4. Discussion

*PvCSP* is the main sporozoite surface protein. It is expressed during the pre-erythrocyte phase and is important for sporozoites to move and invade human hepatocytes. This protein is highly immunogenic and is considered to be a prime vaccine candidate [14]. The three *PvCSP* genotypes are globally distributed, with VK210 being the most common [16]. In this study, VK210 was the most prevalent variant during both study periods (94.0% in the first period and 87.3% in the second period). Our findings showed that these two variants of this gene changed over time. In 2014–2016, 12.7% of the isolates had the VK247 type, up from 5.4% in 2006–2007. A study conducted in Thailand [22] showed that the frequency of the VK247 variant increased over time. This increase may be caused by the varying susceptibilities of mosquito vectors to the VK247 type, host immune selection, or sampling bias [23]. In *P. vivax* malaria-endemic countries, VK210 and VK247 have been found at different frequencies. Isolates collected from the Thai–Myanmar border revealed a dominance of parasites with the VK210 genotype in all the sample sets analyzed [22], a finding noted in other studies in Thailand (93.4%) [24], Pakistan (85.5%) [25], and Guyana (92.0%) [26]. In contrast to previous studies [27,28], a lower frequency of the VK247 genotype was observed in the isolates under this study. *PvCSP* allelic dominance appears to be influenced by variations in parasite transmission, vectorial competence, immune responses, and treatment responses in different endemic areas [23,29,30]. Therefore, these factors should be considered when developing a CSP vaccine. Based on size polymorphisms, 14 allelic variants of *PvCSP* were found, which is consistent with the 17 variants previously observed on the Thai–Myanmar border [22]. However, a study conducted in India reported three variants [31]. Variations in *PvCP* allelic types may be explained by the influence of the induced immune response on a particular allelic variant that infects a person [32].

*PvMSP-3* is a multigene family located on chromosome 10. Among this family, *PvMSP-3α* and *PvMSP-3β* have become genetic markers in *P. vivax* epidemiological studies around the world and are being considered as potential vaccine targets [17]. In this study, three distinct PCR-based types (A, B, and C) were observed. This result is consistent with the allelic variants observed in western and southern Thailand [4], the China–Myanmar border [33], and India [34], and a different band size designated as type D was reported in Pakistan [35]. In Hainan province, China, only types A and B were found [36]. Type A is the most prevalent at each sampling site in the current study. However, it is slightly lower than that found in a previous study of the border areas between Thailand and neighboring countries, which found that type A was 83.3–89.9% more prevalent than the other types. Only 1.8% of the isolates with mixed genotypes were identified using PCR analysis. This proportion is lower than that observed in a recent study carried out in 2014–2016 in diverse geographic areas of Thailand [18]. RFLP of *PvMSP-3α* revealed 28 allelic variants in the first period, of which 25 were found in isolates from Sai Yok, and 23 were found in isolates from Mae Sot. Moreover, the number of allelic variants between Mae Sot isolates in the two time periods varied considerably (14 alleles in 2014–2016 compared with 23 alleles in 2006–2007). This might be due to the differences in sample sizes between the periods. In contrast to our study, fewer allelic variants were reported in Thailand (12 alleles in 2003 and 14 alleles in 2011) [10,32]. A recent study carried out in Thailand showed 40 *PvMSP-3α* allelic variants [18]. Other endemic areas, such as India [37] and Myanmar [38], have also reported similar observations of high diversity. The *PvMSP-3α* genetic diversity in isolates collected in 2006–2007 and 2014–2016 was 10.1% and 14.6%, respectively. An increase in genetic diversity over time was also reported in a study conducted in Thailand [18]. The observed changes in allelic diversity over time can be attributed to the presence of different mosquito species that can transmit specific parasite types in a given area at different times or seasons [39]. Comparing the patterns of allelic variants, at least seven alleles were found to be the same as those found in earlier studies [18,40] conducted in Thailand. This suggests that similar *PvMSP-3α* alleles are distributed among the parasites in the country.

For the *PvMSP-3β* gene, three major types (A, B, and C) and 16 isolates of multiple-genotype infections were found. The three types were also found in northwest Thailand and along the Thai–Myanmar border [18,19]. In contrast, a study conducted in southern Thailand found only types A and B [40]. Our study showed that type A was more prevalent in the earlier samples collected from Sai Yok and Mae Sot, whereas in the isolates from Mae Sot in 2014–2016, types A and B were equally prevalent. A report from the Thai border areas [18] showed a higher frequency of type A. In western Thailand, type B was present in 60.4% of the isolates [32]. From all the samples analyzed for *PvMSP-3β*, only one the type C harboring isolate was found in Mae Sot. This type has been previously reported in Mae Sot isolates [19]. Based on PCR detection, mixed infection in *PvMSP-3β* was found in 15 samples (6%) in the first period, which is three times higher than that in southern Thailand [40]. However, 19.1% of mixed infections were found on the Thai–Myanmar border [18]. The PCR-RFLP of *Pst I* showed that 36 alleles of 248 isolates (14.5%) and 20 alleles of 80 isolates (25%) were identified in the first and second periods of our study, respectively. Similar to *PvMSP-3α*, isolates from the second period showed increased genetic diversity for *PvMSP-3β*. Our analysis indicated a difference in the *PvMSP-3β* allelic patterns between Sai Yok (29 alleles) and Mae Sot (22 alleles). The different structures of the parasite populations at these two sites could be due to sampling bias or dissimilar epidemiological settings. Some of our study’s allelic patterns were similar to those found in earlier studies [18,40], indicating that certain parasite genotypes are distributed across many parts of the country. Frequent population movement may contribute to the distribution of various parasite genotypes across the country. Restriction analysis of *PvMSP-3β* revealed 43 (18.5%) and 10 (12.5%) multiple-genotype infections in the first and second periods, respectively. Relapse and early gametocytogenesis, two biological characteristics of the *P. vivax* parasite, might be the cause of the infection’s high multiplicity [41].

## 5. Conclusions

This study found a relatively high degree of genetic polymorphism in *PvCSP* and *PvMSP-3*, with low rates of multiple-genotype infections. *PvMSP-3* exhibited an increase in allelic variants during the second period; however, *PvMSP-3α* showed a significant difference in allelic variant frequency between the two periods. The proportion of the two variants of the *PvCSP* genotype (VK210 and VK247) changed over time, with an increase in the proportion of VK247 genotypes observed during the second sampling period. The results of this study will help in the understanding of the genetic structure of *P. vivax* and the control of the malarial parasite at the Thai–Myanmar border.

## Figures and Tables

**Table 1:** The proportion of *PvCSP* from the first (2006–2007) and second (2014–2016) periods.

	First Period ^1^			Second Period
	Sai Yok	Mae Sot	Total	Mae Sot
	N %	N %	N %	N %
VK210	160 (92.5)	136 (95.8)	296 (94.0)	89 (87.3)
VK247	11 (6.4)	6 (4.2)	17 (5.4)	13 (12.7)
Mixed	2 (1.2)	0 (0.0)	2 (0.6)	0 (0.0)
Total	173 (100.0)	142 (100.0)	315 (100.0)	102 (100.0)

^1^ Significant difference between first and second period (*p* = 0.012).

**Table 2:** The proportion of *PvMSP-3α* from the first (2006–2007) and second (2014–2016) periods.

First Period ^1^					Second Period		
	Sai Yok		Mae Sot ^3^		Mae Sot		
	N	%	N	%		N	%
Type A (77.3 %)	136	78.6	78	75	Type A (78.1%)	75	78.1
A1	8	4.6	3	2.9	A3	4	4.2
A2	1	0.6	0	0.0	A4	1	1.0
A3	15	8.7	4	3.8	A5	3	3.1
A4	5	2.9	2	1.9	A7	1	1.0
A5	8	4.6	8	7.7	A8	12	12.5
A6	5	2.9	0	0.0	A10	2	2.1
A7	1	0.6	1	1.0	A11	31	32.3
A8	31	17.9	11	10.6	A12	5	5.2
A9	7	4.0	8	7.7	A13	12	12.5
A10	2	1.2	0	0.0	A18	1	1.0
A11	1	0.6	11	10.6	A19	1	1.0
A12	11	6.4	9	8.7	A22	2	2.1
A13	4	2.3	2	1.9	Type B (21.9%)	21	21.9
A14	2	1.2	2	1.9	B2	7	7.3
A15	0	0.0	3	2.9	B3	14	14.6
A16	1	0.6	3	2.9	Total	96	100.0
A17 *	2	1.2	2	1.9	Pattern (14, 14.6%)	14	
A18	27	15.6	7	6.7			
A19 *	4	2.3	1	1.0			
A20	1	0.6	0	0.0			
A21	0	0.0	1	1.0			
Type B (7.9 %)	13	7.6	9	8.7			
B1	6	3.5	5	4.8			
B2	6	3.5	2	1.9			
B3	1	0.6	2	1.9			
Type C (13%)	19	11.0	17	16.3			
C1	17	9.8	15	14.4			
C2	2	1.2	1	1.0			
C3	0	0.0	1	1.0			
M ^2^ (1.8%)	5	2.9	0.0	0.0			
Total	173	100.0	104	100.0			
Pattern (28, 10.1 %)	25		23				

M: Mixed alleles. * M: Mixed genotype infection after RFLP analysis. **^1^** The overall statistically significant differences in allele frequencies between the two periods (*p* < 0.001). **^2^** Significant difference between first and second period with type M (*p* = 0.002) and type B (*p* < 0.001). **^3^** Significant differences between the Mae Sot isolates from the two time periods (*p* = 0.009).

**Table 3:** The proportion of *PvMSP-3β* from the first (2006–2007) and second (2014–2016) periods.

First Period					Second Period		
	Sai Yok		Mae Sot		Mae Sot		
	N	%	N	%		N	%
Type A (63.4%)	102	63.0	55	66.3	Type A (51.2%)	41	51.2
A1	32	19.4	16	19.3	A1	14	17.5
A2	2	1.2	0	0.0	A3	2	2.5
A3	0	0.0	4	4.8	A4	1	1.3
A4	3	1.8	1	1.2	A7 *	8	10.0
A5 *	3	1.8	0	0.0	A10	2	2.5
A6 *	0	0.0	1	1.2	A11	2	2.5
A7 *	11	6.7	7	8.4	A12	1	1.3
A8 *	5	3	0	0.0	A14	10	12.5
A9	0	0.0	1	1.2	A23	1	1.3
A10	1	0.6	2	2.4	Type B (47.5%)	38	47.5
A11	23	13.9	9	10.8	B1	2	2.5
A13 *	2	1.2	0	0.0	B2	1	1.3
A14	1	0.6	2	2.4	B3	2	2.5
A15	2	1.2	1	1.2	B4	22	27.5
A16	4	2.4	7	8.4	B5	2	2.5
A17	3	1.8	1	1.2	B6	1	1.3
A18 *	2	1.2	1	1.2	B7 *	2	2.5
A19	1	0.6	0	0.0	B8	1	1.3
A20 *	1	0.6	0	0.0	B9	2	2.5
A21 *	3	1.8	0	0.0	B12	3	3.8
A22 *	3	1.8	0	0.0	Type M (1.3%)	1	1.3
A24	0	0.0	2	2.4	Total	80	100.0
Type B (30.2%)	52	30.3	23	27.7	Pattern (20, 25%	20	
B1	2	1.2	3	3.6			
B2	2	1.2	0	0.0			
B3	4	2.4	1	1.2			
B4	27	16.4	14	16.9			
B5	3	1.8	0	0.0			
B6 *	1	0.6	0	0.0			
B7 *	2	1.2	0	0.0			
B8	1	0.6	0	0.0			
B9	5	3.0	2	2.4			
B10 *	0	0.0	2	2.4			
B11	0	0.0	1	1.2			
B12	4	2.4	0	0.0			
B13 *	1	0.6	0	0.0			
Type C (0.4 %)	0	0.0	0	1.2			
C1	0	0.0	1	1.2			
Type M (6 %)	11	6.7	4	4.8			
Total	165	100.0	83	100.0			
Pattern (36, 14.5%)	29		22				

M: Mixed alleles. * Mixed genotype infection after RFLP analysis.

## Data Availability

Data will be made available upon request.
